# Transcriptome Profiling Reveals Indoxyl Sulfate Should Be Culpable of Impaired T Cell Function in Chronic Kidney Disease

**DOI:** 10.3389/fmed.2020.00178

**Published:** 2020-05-06

**Authors:** Fangfang Xiang, Xuesen Cao, Bo Shen, Xiaohong Chen, Man Guo, Xiaoqiang Ding, Jianzhou Zou

**Affiliations:** ^1^Department of Nephrology, Zhongshan Hospital, Fudan University, Shanghai, China; ^2^Shanghai Key Laboratory of Kidney and Blood Purification, Shanghai, China; ^3^Shanghai Institute of Kidney and Dialysis, Shanghai, China; ^4^Shanghai Medical Center for Kidney, Shanghai, China

**Keywords:** T cell, indoxyl sulfate, aryl hydrocarbon receptor, chronic kidney disease, RNA-sequencing

## Abstract

**Introduction:** Chronic inflammation and immune system dysfunction have been evaluated as major factors in the pathogenesis of chronic kidney disease (CKD), contributing to the high mortality rates observed in these populations. Uremic toxins seem to be the potential “missing link.” Indoxyl sulfate (IS) is one of the protein-bound renal toxins. It participates in multiple pathologies of CKD complications, yet its effect on immune cell has not been studied. This study aimed to explore the genome-wide expression profile in human peripheral blood T cells under stimulation by IS.

**Methods:** In this study, we employed RNA-sequencing transcriptome profiling to identify differentially expressed genes (DEGs) responding to IS stimulation in human peripheral T cells *in vitro*. Flow cytometry and western blot were used to verify the discovery in RNA-sequencing analysis.

**Results:** Our results yielded a total of 5129 DEGs that were at least twofold up-regulated or down-regulated significantly by IS stimulation and half of them were concentration-specific. Analysis of T cell functional markers revealed a quite different transcription profile under various IS concentration. Transcription factors analysis showed the similar pattern. Aryl hydrocarbon receptor (AhR) target genes CYP1A1, CYP1B1, NQO1, and AhRR were up-regulated by IS stimulation. Pro-inflammatory genes TNF-α and IFN-γ were up-regulated as verified by flow cytometry analysis. DNA damage was induced by IS stimulation as confirmed by elevated protein level of p-ATM, p-ATR, p-BRCA1, and p-p53 in T cells.

**Conclusion:** The toxicity of IS to T cells could be an important source of chronic inflammation in CKD patients. As an endogenous ligand of AhR, IS may influence multiple biological functions of T cells including inflammatory response and cell cycle regulation. Further researches are required to promulgate the underling mechanism and explore effective method of reserving T cell function in CKD.

## Introduction

Chronic inflammation and immune system dysfunction have been evaluated as major factors in the pathogenesis of chronic kidney disease (CKD), contributing to the high mortality rates observed in these populations. As a main component of cellular immunity, T cells play a leading role in defense of pathogens, immune homeostasis and immune surveillance. The impact of uremia on the immune system has been previously studied in end-stage renal disease (ESRD) patients. Betjes et al. (1, 2) described a decline of T cell numbers especially naive T cells with an increased susceptibility to activation-induced apoptosis, expansion of terminally differentiated T cells with highly secretion of proinflammatory cytokines and lack of adequate antigen-specific T cell differentiation which may be the probable cause of high risk of infection in these patients. Uremic toxins have been suggested as a potential “missing link” between CKD and cardiovascular disease (CVD) since higher CVD risk in these patients cannot be sufficiently explained by classic factors. However, there are few studies on the mechanism of T cell dysfunction caused by uremic toxins.

Indoxyl sulfate (IS) is a renal toxin that accumulates in blood of uremic patients with 97.4% bound to serum albumin (3). The serum level of IS in healthy humans is almost undetectable, whereas in uremic patients, it escalates to 236 μg/ml (4). IS plays an important role in the progression of renal disease, CVD, bone metabolism disorders and other complications by promoting oxidative stress and inflammatory response (5). IS also affects T cell differentiation. It has been reported that IS aggravates experimental autoimmune encephalomyelitis by stimulating Th17 differentiation (6) and lessens allergic asthma by regulating Th2 differentiation (7). As one of the endogenous ligands of aryl hydrocarbon receptor (AhR), IS probably make effect through AhR. Emerging evidence suggests that AhR is a key sensor allowing immune cells to adapt to environmental conditions and changes in AhR activity have been associated with autoimmune disorders and cancer (8). However, it remains largely unknown if whether IS or AhR are responsible for the T cell disfunction in uremic patients.

In the past decade, next-generation sequencing technology has emerged as an effective tool to investigate the gene expression profiling of a species under specific conditions. The advantages of speed, precision and high-efficiency performance of RNA sequencing (RNA-seq) encouraged us to explore the genome-wide expression profile in human peripheral blood T cells under stimulation by IS.

## Methods

### Cell Isolation and Activation

Buffy coats from healthy donors were obtained from Zhongshan Hospital, Fudan University. This study has been approved by the Medical Ethics committee of Zhongshan Hospital, Fudan University. Peripheral blood mononuclear cells were separated by density-gradient centrifugation using Ficoll-Paque Plus (GE healthcare Bio-Science, Uppsala, Sweden) and were further processed for separation of T cells using CD3 MicroBeads (MiltenyiBiotec, Auburn, USA). T cells were cultured in RPMI medium (Eurobio, Les Ulis, France) supplemented with 20 IU/mL penicillin, 20 μg/mL streptomycin, and 10% decomplemented FBS (Life Technologies), and stimulated with Dynabeads® T-Expander beads coated with anti-CD3 and anti-CD28 Abs (Life Technologies) at a 1:1 cell/bead ratio in the absence of IL-2 (30 U/ml). Then IS stimulation experiments were conducted by treating T cells with different IS concentration for 96 h.

### RNA Sequencing

Total RNA was extracted from each sample using TRIzol reagent (Invitrogen, Carlsbad, CA, USA) following the manufacturer's protocol. The RNA concentration and purity were checked by OD A260/A280 (>1.8) and A260/A230 (>1.6). The quality and quantity of RNA obtained from each sample was checked using the NanoPhotometer® spectrophotometer (IMPLEN, CA, USA). RNA concentration was measured using Qubit® RNA Assay Kit in Qubit®2.0 Flurometer (Life Technologies, CA, USA). RNA integrity was assessed using the RNA Nano 6000 Assay Kit of the Bioanalyzer 2100 system (Agilent Technologies, CA, USA). A total amount of 3 μg RNA per sample was used as input material for the RNA sample preparations. Sequencing libraries were generated using NEBNext® Ultra TM RNA Library Prep Kit for Illumina® (NEB, USA) following manufacturer's recommendations and index codes were added to attribute sequences to each sample. The clustering of the index-coded samples was performed on a cBot Cluster Generation System using TruSeq PE Cluster Kit v3-cBot-HS (Illumia) according to the manufacturer's instructions. After cluster generation, the library preparations were sequenced on an Illumina Hiseq platform and 125 bp/150 bp paired-end reads were generated.

### RNA-Seq Data Processing

Clean reads were obtained by removing reads containing adapter, reads containing ploy-N and low-quality reads from raw data. Reference genome and gene model annotation files were downloaded from genome website directly. Index of the reference genome was built using Hisat2 v2.0.5 and paired-end clean reads were aligned to the reference genome using Hisat2v2.0.5. The read counts of each transcript were normalized to the length of the individual transcript and to the total mapped read counts in each sample and were expressed as FPKM. Differential expression analysis of two groups was performed using the DESeq2 R package (1.16.1). *P*-values were adjusted using the Benjamini and Hochberg's approach for controlling the false discovery rate. In the analysis, a criterion of |log_2_(fold-change)| > 0 and an adjusted *P* < 0.05 were assigned as differentially expressed. Hierarchical clustering was utilized to present the selected significant down-regulated and up-regulated genes. The cluster Profiler R package was used to perform the gene ontology (GO) enrichment analysis (http://www.geneontology.org). Kyoto Encyclopedia of Genes and Genomes (KEGG, https://www.genome.jp/kegg) and Reactome (https://reactome.org) pathway analysis were performed to understand the function and interactions among differentially expressed genes.

### Flow Cytometry Analysis

After culture, purified T cells were washed twice in PBS with 1% FBS and subsequently stained for 30 min at 4°C with the following fluorescein-conjugated monoclonal antibodies: human anti-CD3-PE (Biolegend, San Diego, CA), anti-CD4-APC (Biolegend, San Diego, CA), anti-CD8-PerCP/CY5.5(Biolegend and San Diego, CA). Stained cells were resuspended for 30 min at 4°C in Cytofix/Cytoperm fixation/permeabilization solution (Thermo Fisher Scientific, Waltham, MA, USA), according to the manufacturer's instructions. Once permeabilized, cells were washed twice and stained for intracellular cytokines with the following mAbs: human anti-IFNγ-FITC (Biolegend, San Diego, CA), anti–TNF-α-650^TM^ (Biolegend, San Diego, CA). A total of 200,000 events were acquired by the BD LSRFortessa™ flow cytometer (BD Bioscience, San Jose, CA, USA). FlowJo v10.1 software (Tree Star, Ashland, OR, USA) was used for date analysis.

### Western Blot Analysis

The cells were washed twice with cold PBS and then lysed in RIPA buffer supplemented with complete EDTA-free Protease Inhibitor Cocktail (Roche Applied Science, Mannheim, Germany) and PhosStop Phosphatase Inhibitor Cocktail (Roche Applied Science) on ice for 30 min. The cell lysates were sonicated five times for 10 s each and centrifuged at 11,000 g for 30 min at 4°C. The supernatants were subsequently collected. Protein concentrations were measured using a BCA protein assay kit (Pierce, Inc., Rockford, IL).

Protein samples of 50 μg were separated by sodium dodecyl sulfate polyacrylamide gel electrophoresis and transferred to a PVDF membrane (Millipore, Inc.). Immunoblotting was conducted using rabbit anti-phospho-ATR (Ser 428) antibody, rabbit anti-phospho-BRCA1 (Ser 1524) antibody, rabbit anti-phospho-ATM (Ser1981) antibody, mouse anti-phospho-p53 (Ser15) antibody, rabbit anti-AhR antibody, anti-rabbit IgG antibody, anti-mouse IgG antibody. All antibodies were purchased from Cell Signaling Technology, Inc. (1:1,000). The ECL-enhanced chemiluminescence system (Amersham) was used for detection. Images were quantified by Image J1.34 Software. The intensity of band was normalized to the GAPDH.

### Statistical Analysis

Data were reported as mean ± SD. Statistical analysis was performed using the GraphPad Prism5 software. The one-way ANOVA was used for multiple group comparisons. The paired Student's *t*- test was used for a single comparison between two groups, and the non-parametric *t*-test was also chosen if the sample size was too small and not fit Gaussian distribution.

## Results

### Purity of T Cells and RNA-Seq Profiling Analysis

**Figure 1:**
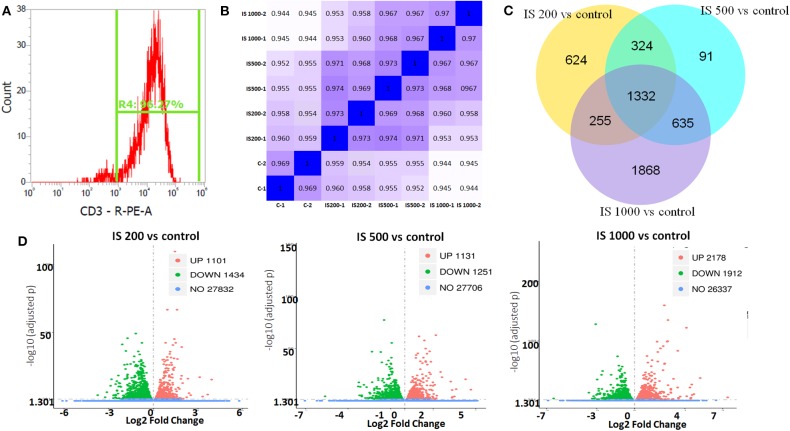
T cell purity and global patterns of RNA-seq profiling. **(A)** The purity of T cells confirmed by flow cytometry; **(B)** Hierarchical clustering based on Pearson correlation coefficients among samples in IS groups; **(C)** Volcano plot showed the numbers of up and down regulated DEGs in each IS groups compared to the control group; **(D)** Veen figure showed the common and different DEGs in each IS groups.

The purity of T cells was >97%, as confirmed by flow cytometry (Figure 1A). In this study, after filtered adapter and low-quality reads, about 44.25–62.64 million clean reads were obtained for all samples (Table S1). Hierarchical clustering based on Pearson correlation coefficients showed high correlation (0.969–1.00) among samples in each group and IS stimulation groups were distinctly separated from control group (Figure 1B).

**Figure 2:**
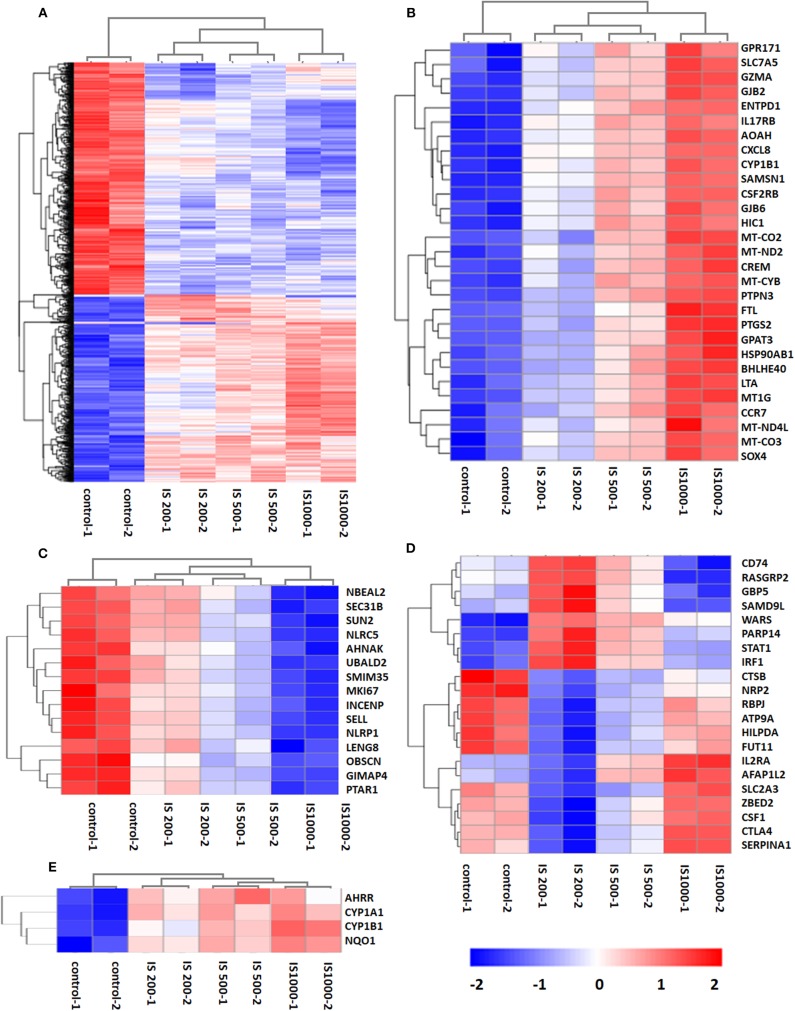
Hierarchical cluster analysis of patterns of DEGs. **(A)** 1,332 common DEGs in all IS groups compared with the control groups; **(B)** 29 DEGs up-regulated by IS simulation in a concentration dependent manner; **(C)** 15 DEGs down-regulated by IS stimulation in a concentration dependent manner; **(D)** 20 DEGs up-regulated or down-regulated at 200 μM but reversely regulated when IS concentration was higher; **(E)** AhR target genes CYP1A1, CYP1B1, NQO1, and AhRR were up-regulated by IS stimulation.

There were 5129 DEGs that were at least twofold up-regulated or down-regulated significantly by IS stimulation and half of them were concentration-specific. Compared with the control group, there were 2535 DEGs in the group treated by 200 μM IS, of which 1101 DEGs were up-regulated and 1434 DEGs were down-regulated. Group treated by 500 μM IS had 2382 DEGs, of which 1131 DEGs were up-regulated and 1251 DEGs were down-regulated. Group treated by 1,000 μM IS had 4090 DEGs, of which 2178 DEGs were up-regulated and 1912 DEGs were down-regulated (Figure 1C). 1332 were common DEGs in all groups treated by various concentrations of IS compared with the control groups (Figure 1D). Of all common 1332 DEGs, 29 DEGs were up-regulated and 15 DEGs were down-regulated in a concentration dependent manner. **21** DEGs were up-regulated or down-regulated at 200μM but reversely regulated when IS concentration was higher. These DEGs were listed in Figure 2.

### Functional Categories and Enriched Pathways by RNA-Seq Analysis

We firstly focused on the T cell functional markers including cluster of differentiation, cytokine and cytokine receptor. Two hundred and seventy two genes were screened (Table S2) and 87 differently expressed genes were found under filtering conditions (corrected *P* < 0.0001 and FPKM>1), which were listed in Table 1. These genes were involved in T cell activation, adhesion and signal transduction. 19 genes including LTA, LTB, CXCL8, and CCR7 were up-regulated at IS concentration of 200 μM. Twenty genes including TNF-α, IFN-γ and CD40L were up-regulated when IS concentration were higher. 41 genes including IL2, CD28, PD1, and CTLA4 were down-regulated at IS concentration of 200 μM; some of them returned to normal or even up-regulated when IS concentration were higher. The effects of IS stimulation on AhR activation were shown in Figure 2E. mRNA levels of AhR target genes, CYP1A1, CYP1B1, NQO1, and AhRR were increased by IS stimulation, indicating a transcriptionally active form of AhR.

**Table 1:** Differentially expressed genes of T cell functional markers on IS stimulation.

	**Gene name**
Up-regulated at 200 μM	LTA, LTB, CXCL8, IL17RB, IL7R, IL9R, CSF2RB, TNFSF8, SIRPG, CCR7, CCR8, CD38, ENTPD1, CD48, ITGA4, CD74, CD96, SLC7A5, TNFSF10
Down-regulated at 200 μM	CD28, CTLA4, PDCD1, IL2, IL2RB, IL4R, DPP4, LAG3, IL1RN, IL4I1, IL21, SEMA7A, ITGAX, TNFRSF1B, TNFRSF4, ADAM8, SELPLG, DDR1, ITGB2, CD200, IL10RA, CD22, IGF1R, CD24, CD276, TNFRSF8, CD3E, CD4, CD47, ITGA3, ITGA5, CD5, CD6, SELL, CD7, CD82, THY1, ADGRE5, CD99, TNFRSF13C, SLAMF1
Up-regulated at 500 μM	IL2RA, TNF, CD40LG, IL23R, IL13, IL18R1, HMMR, CD2, TFRC, CD84, LTA, LTB, CXCL8, IL17RB, IL7R, IL9R, CSF2RB, TNFSF8, SIRPG, CCR7, CCR8, CD38, ENTPD1, CD48, ITGA4, CD74, CD96, SLC7A5, TNFSF10
Down-regulated at 500 μM	IL31RA, CD37, CD28, CTLA4, PDCD1, IL2, IL4R, DPP4, LAG3, IL1RN, IL4I1, IL21, ITGAX, TNFRSF4, ADAM8, SELPLG, DDR1, ITGB2, CD200, IL10RA, CD22, IGF1R, CD24, CD276, CD3E, CD4, CD47, ITGA3, ITGA5, CD5, CD6, SELL, CD7, CD82, THY1, ADGRE5, CD99, TNFRSF13C
Up-regulated at 1,000 μM	IFNG, FASLG, IL26, TNFRSF9, PTPRJ, CXCR6, CD226, CD69, CD9, LTA, LTB, CXCL8, IL17RB, IL9R, CSF2RB, TNFSF8, SIRPG, CCR7, CCR8, CD38, ENTPD1, CD48, ITGA4, CD96, SLC7A5, TNFSF10, IL2RA, TNF, CD40LG, IL23R, IL13, IL18R1, HMMR, CD2, TFRC, CD84, CTLA4, IL2RB, SEMA7A
Down-regulated at 1,000 μM	IL11RA, MUC1, CD8B2, IL3RA, FGFR1, CD74, IL31RA, CD37, CD28, IL2, IL4R, DPP4, LAG3, IL1RN, IL4I1, IL21, ITGAX, TNFRSF4, ADAM8, SELPLG, ITGB2, CD200, IL10RA, CD22, IGF1R, CD24, CD3E, CD4, CD47, ITGA3, ITGA5, SELL, CD7, THY1, ADGRE5, CD99, TNFRSF13C, TNFRSF1B

**Figure 3:**
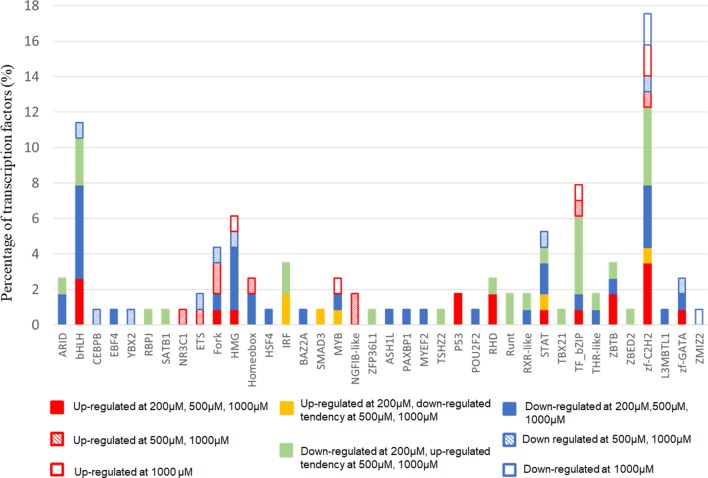
The distribution of TF families. The x-axis represents different TF families (gene names were presented directly if there was only one TF in this TF family), the y-axis represents the percentage of corresponding TF family in total differentially expressed TFs.

**Figure 4:**
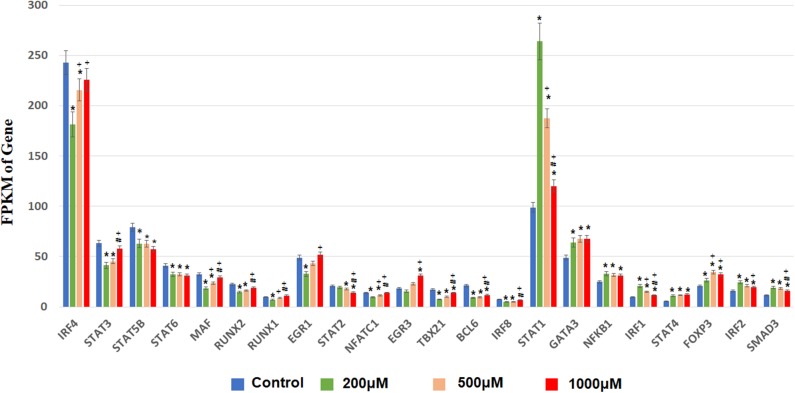
FPKM of TFs involved in T cell differentiation in each IS group. The x-axis represents FPKM, the y-axis represents gene name and IS concentration. *corrected *P* < 0.05 compared with control group; ^+^corrected *P* < 0.05 compared with IS 200 μM group; ^#^corrected *P* < 0.05 compared with IS 1,000 μM group.

Next, we focused on transcription factor (TF) responsive to IS stimulation. TF analysis of differentially expressed genes were extracted directly from the AnimalTFDB database. 114 TFs differentially expressed when compared to the control group under filtering conditions (corrected *P* < 0.0001). The three mostly altered TF families were zf-C2H2, bHLH, and TF-bZIP separately (Figure 3). Most TFs were differentially expressed at IS concentration of 200 μM, of which 24 TFs were up-regulated and 64 TFs were down-regulated. With a similar pattern of T cell functional markers, many of these TFs were diversely regulated when IS concentration was higher. Eighteen more TFs were differentially expressed when IS concertation raised to 500 μM and 9 TFs were only differentially expressed at IS concentration of 1,000 μM. Myc, BHLHE40, SOX4, CREM, and HIC1 were up-regulated in a concentration dependent manner. Several major regulators of T cell differentiation were also affected by IS stimulation. STAT1, STAT4, NF κ B1 (P50/P105), GATA3, Foxp3, Smad3, and IRF2 were significantly up-regulated. STAT3, STAT5B, STAT6, T-bet, MAF, Runx2, Runx1, BCL6, and NFATC1 were significantly down-regulated at IS concentration of 200 μM. Except for STAT5B and STAT6, these TFs were relatively up-regulated at 500 μM or 1,000 μM. STAT2 was significantly down-regulated when IS concentration was higher than 500 μM (Figure 4).

**Figure 5:**
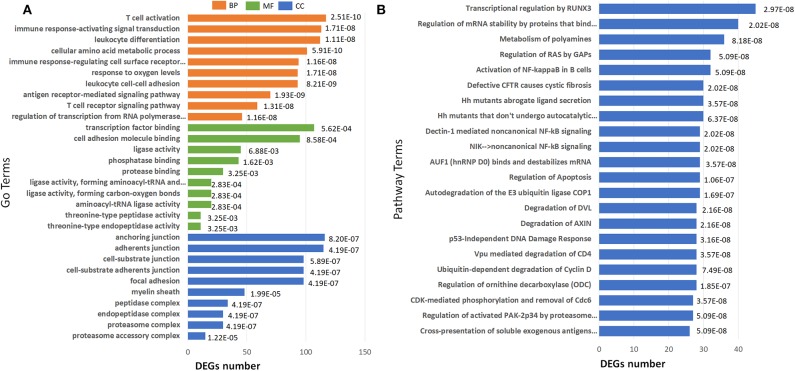
Barplots of significantly enriched terms. **(A)** GO enrichment and **(B)** Reactome enrichment terms under corrected *P* < 0.001. The x-axis represents differentially expressed genes number, the y-axis represents GO or Reactome pathway terms; the numbers in the plot are the corrected *P*-values.

GO term enrichment analysis of the 2040 DEGs in IS 500 μM group revealed 182 significantly enriched GO terms under filtering conditions (corrected *P* < 0.001). The top 10 GO terms of the three aspects [Biological Process (BP), Molecular Function (MF) and Cellular Component (CC)] were shown in Figure 5A. The enriched GO terms on BP and MF were mainly related to immune function (e.g., “T cell activation,” “antigen receptor-mediated signaling pathway,” “leukocyte cell-cell adhesion,” “leukocyte differentiation”), gene expression (e.g., “regulation of transcription from RNA polymerase II promoter in response to stress”). The top GO terms on CC were proteasome complex and focal adhesion. Reactome pathway analysis revealed lots of enriched terms concerning cell cycle especially DNA damage in the top 20 (e.g., “Autodegradation of the E3 ubiquitin ligase COP1,” “p53-Independent DNA Damage Response”). The top 20 Reactome pathway terms were shown in Figure 5B.

### Effects of IS on Inflammation and DNA Damage in Human Peripheral T Cells

**Figure 6:**
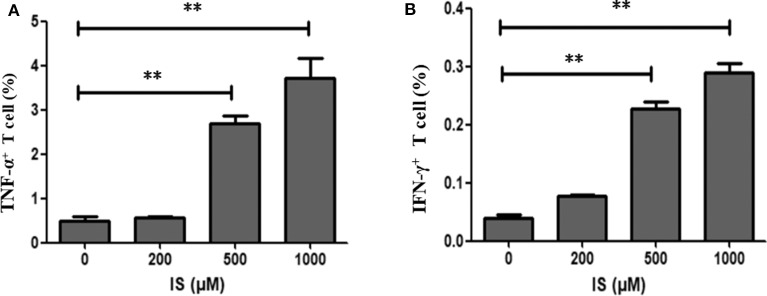
Secretion of TNF–α and IFN-γ in T cells by IS intervention. **(A)** Secretion of TNF–α was significantly elevated in T cells in IS 500 μM group and IS 1,000 μM group; **(B)** Secretion of IFN-γ was significantly elevated in T cell in IS 500μM group and IS 1,000 μM group. **P < 0.001 compared with the control group.

To validate RNA-seq results, flow cytometry was performed on two most common pro-inflammatory factors TNF-α and IFN-γ. After 4 days of stimulation, secretion of TNF–α and IFN-γ were significantly elevated in T cells (Figure 6).

**Figure 7:**
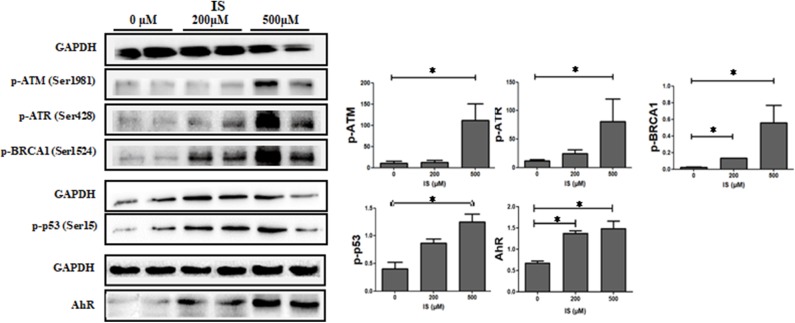
Expression of DDR protein in T cells by IS intervention. Protein level of p-ATM, p-ATR, p-p53 and p-BRCA1 were significantly higher in IS 500 μM group compared with the control group. Protein level of p-BRCA1 were also increased in IS 200 μM group. AhR were also up-regulated in IS 200 μM and IS 500 μM group. *P < 0.05 compared with the control group.

To verify the pathway enrichment results that IS induced DNA damage in T cells, proteins related to DNA damage response (DDR) were tested by western blot. Protein level of p-ATM, p-ATR, p-p53, and p-BRCA1 were significantly higher in IS intervention group and so was AhR (Figure 7).

## Discussion

The RNA-seq provided a rapid and cost-effective way to obtain all transcribed mRNA expression profiles within a specific period. Here we studied the transcriptome of human peripheral T cells to understand gene expression profiles under IS stimuli. Interestingly, we found quite different transcription profile under various concentration of IS stimulation indicating IS may have different effects on T cell function at distinct stages of CKD. As reported earlier, serum level of IS averages 35.5 mg/ml and escalate to maximum value of 236 mg/ml in dialysis patients (4, 9). In the current study, the intervention concentration of IS covered the medium and high serum IS level in uremic patients.

By screening transcription of T cell functional markers including cluster of differentiation, cytokine and cytokine receptor, we found lots of pro-inflammatory genes were up-regulated such as TNF-α, IFN-γ, CD40L, and CXCL8. Flow cytometry further proved that IS elevated the secretion of TNF -α and IFN-γ in T cells. In addition, IS down regulated CD28 expression, which could be vital since CD4^+^CD28^−^ T cell has been well-proved to be closely related to chronic inflammation and clinical CVD events in CKD patients (10). CD7 and CD26, the two factors reported been down-regulated in chronic inflammatory diseases (11, 12), were also down-regulated by IS intervention. Many molecules known as immune checkpoint inhibitor, which activating in evolving immune activation cascade and contributing inhibitory signals to dampen an overexuberant response, were down-regulated, including PD1, CTLA4, LAG3, and CD200 (13–15). Down-regulation of these genes could aggravate inflammation. These results highly suggested that the toxicity of IS to T cells could be an important source of chronic inflammation in CKD or uremic patients. Many factors would contribute to chronic inflammatory status in CKD, including increased production of proinflammatory cytokines, oxidative stress, acidosis, altered metabolism of adipose tissue and even some treatment *per se* such as hemodialysis (16). Immune cells activated by uremic milieu produce more proinflammatory cytokines and aggravate the inflammation condition as a vicious circle. The most well-documented studies were that patients with ESRD typically had an expansion of proinflammatory CD4^+^CD28^−^ T cell and CD14^+^CD16^++^ monocyte populations, which were considered to be novel, non-traditional cardiovascular risk factors (2). Besides cytokines, retention of uremic toxins should be the key mechanism that underlie the generation of oxidative stress and inflammation. As matter of fact, the crosstalk between gut microbiota and CKD has become a new focus for studying the mechanism of inflammation in these patients. IS and p-cresyl sulfate, generated by protein fermentation in intestine, are potential candidates since they were not only associated with CKD progression but also related to poor prognosis in ESRD patients (17, 18). Our study shed a light into the mechanism of immune disturbance in CKD patients.

As an endogenous ligand of AhR, IS is functioning through AhR along with many other uremic toxins, such as indole-3-acetic acid and indoxyl-β-D glucuronide (19, 20). AhR is a ligand-activated transcription factor and is involved in the regulation of multiple cellular pathways such as inflammatory responses, cell cycle regulation and hormone signaling (21). Physiological functions of AhR may require tightly controlled and transient signaling, and sustained AhR signaling may underlie pathological responses (22). Accumulation of IS in CKD patients could cause prolonged AhR activation; it further leading to a pathological change. Recently, a clinical study confirmed that CKD patients displayed a strong AhR-activating potential, which is not only strongly correlated with serum IS level but also correlated with CVD risk (23). In the current study, the expression of AhR protein and known AhR-regulated genes such as CYP1A1, CYP1B1, NQO1, and AhRR were up-regulated, indicating a transcriptionally active form of AhR, which was consistent with the previous study (7). When analyzing transcription factors, we found some critical TFs such as STAT1, STAT3, IRF4 were differentially expressed at IS concentration of 200 μM, but were diversely regulated when IS concentration was higher. In addition, the plasma IS concentration of CKD patients is far beyond the scope of this experimental design, and it remains a question how TFs react at lower IS concentration. It seems that the effect of IS on T cells are quite different depending on various IS concentration. At present, we cannot fully understand the mechanism of TFs rebound. We speculate this may be related to the characteristics of AhR function, since lots of previous studies have shown that activated AhR could function oppositely by different kinds of ligand or even one ligand in different concentrations (24, 25). It is worth noting that in GO and Reactome analysis, we found a lot of enriched items were concerning cell cycle especially DNA damage. Western blot also confirmed that the expression of DDR proteins including p-ATM, p-ATR, p-p53, and p-BRCA1 were up-regulated under IS stimulation. Therefore, we suggest that IS may cause DNA damage, which could further lead to T cell senescence. Notably, T cell senescence has been considered a major contributor of inflammation and crucial mechanism of complications in CKD (11, 26, 27), thus it should be paid enough attention that IS may lead to DNA damage. We can't get answer here whether IS directly leads to DNA damage through AhR, or indirectly through inflammation or oxidative stress. But the first case is reasonable since it has been well-proved that activation of AhR could directly cause DNA damage in other settings (28, 29).

Our study had several limitations. First, this study was based on IS effect on T cells from healthy donors. Since inflammatory response in T cells of CKD patients could be different compared to the response in healthy T cells treated with IS, further research focused on patients' T cells should be conducted to providing better understanding of effect of IS on T cell function in CKD. Secondly, this research only presented an image of influence by IS to T cells, studies with the aim of understanding molecular mechanism are needed.

In conclusion, our study shows that the toxicity of IS to T cells could be an important source of chronic inflammation in CKD patients. As an endogenous ligand of AhR, IS may participate in multiple cellular pathways such as inflammatory response and cell cycle regulation, which are closely related to impaired T cell function in CKD patients. We hope that this study will encourage other laboratories around the world to get more in-depth knowledge of the molecular mechanism of uremia associated immune dysfunction and make efforts to improve the clinical prognosis of CKD patients.

## Data Availability Statement

The datasets generated for this study can be found in the SRA, PRJNA599948.

## Ethics Statement

The studies involving human participants were reviewed and approved by Ethical Committee, Zhongshan Hospital, Fudan University. The patients/participants provided their written informed consent to participate in this study.

## Author Contributions

FX conducted the experiment and drafted the paper. JZ and XCa designed the experiments. MG and XCh provided technical support. XD and BS revised the manuscript. All authors read and approved the final manuscript.

## Conflict of Interest

The authors declare that the research was conducted in the absence of any commercial or financial relationships that could be construed as a potential conflict of interest.

## Supplementary Material

Table S1 Clean reads for all samples.

Table S2 T cell markers screened for DEGs.
